# 4-(3-Methoxy­phen­yl)-2,6-dimethyl­cyclo­hex-3-enecarboxylic acid

**DOI:** 10.1107/S1600536810018544

**Published:** 2010-05-26

**Authors:** Songwen Xie, Dannette A. Nusbaum, Holly J. Stein, Maren Pink

**Affiliations:** a Department of Natural, Information, and Mathematical Sciences, Indiana University Kokomo, Kokomo, IN 46904–9003, USA; b Indiana University Molecular Structure Center, Indiana University, Bloomington, IN 47405–7102, USA

## Abstract

The racemic title compound, C_16_H_20_O_3_, was synthesized to study the hydrogen-bonding inter­action of the two enanti­o­mers in the solid state. In the crystal structure, *R* and *S* pairs of the racemate are linked by pairs of inter­molecular O—H⋯O hydrogen bonds, producing centrosymmetric *R*_2_^2^(8) rings.

## Related literature

For similar compounds in which the racemates also consist of carboxylic acid *RS* dimers, see: Xie *et al.* (2002 ▶, 2007*a* ▶, 2008*a* ▶,*b* ▶). For the structure of the precursor of the title compound, which is achiral and forms hydrogen-bonded dimers, see: Xie *et al.* (2007*b* ▶). The chirality of the title compound is solely generated by the presence of the double bond in the cyclo­hexene ring, see: Xie *et al.* (2004 ▶). For hydrogen-bond motifs, see: Bernstein *et al.* (1995 ▶).
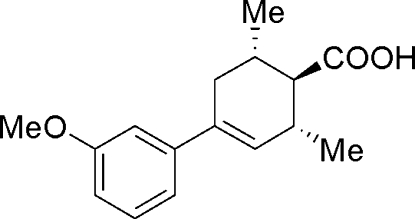


## Experimental

### 

#### Crystal data

C_16_H_20_O_3_
*M* *_r_* = 260.32
Orthorhombic, 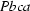

*a* = 11.032 (2) Å
*b* = 7.8423 (17) Å
*c* = 32.140 (8) Å
*V* = 2780.7 (11) Å^3^
*Z* = 8
Synchrotron radiation
λ = 0.44280 Å
μ = 0.05 mm^−1^
*T* = 100 K
0.02 × 0.01 × 0.01 mm

#### Data collection

Bruker Platform goniometer diffractometer
Absorption correction: multi-scan (*SADABS*; Bruker, 2007 ▶; Blessing, 1995 ▶) *T*_min_ = 0.999, *T*_max_ = 1.000
8586 measured reflections
2173 independent reflections
1755 reflections with *I* > 2σ(*I*)
*R*_int_ = 0.050

#### Refinement

*R*[*F* ^2^ > 2σ(*F* ^2^)] = 0.037
*wR*(*F* ^2^) = 0.119
*S* = 1.11
2173 reflections
179 parameters
H atoms treated by a mixture of independent and constrained refinement
Δρ_max_ = 0.17 e Å^−3^
Δρ_min_ = −0.23 e Å^−3^

### 

Data collection: *APEX2* (Bruker, 2007 ▶); cell refinement: *SAINT* (Bruker, 2007 ▶); data reduction: *SAINT*; program(s) used to solve structure: *SIR2004* (Burla *et al.*, 2005 ▶); program(s) used to refine structure: *SHELXL97* (Sheldrick, 2008 ▶); molecular graphics: *SHELXTL* (Sheldrick, 2008 ▶); software used to prepare material for publication: *SHELXTL*.

## Supplementary Material

Crystal structure: contains datablocks global, I. DOI: 10.1107/S1600536810018544/fj2304sup1.cif

Structure factors: contains datablocks I. DOI: 10.1107/S1600536810018544/fj2304Isup2.hkl

Additional supplementary materials:  crystallographic information; 3D view; checkCIF report

## Figures and Tables

**Table 1:** Hydrogen-bond geometry (Å, °)

*D*—H⋯*A*	*D*—H	H⋯*A*	*D*⋯*A*	*D*—H⋯*A*
O2—H2*O*⋯O1^i^	0.94 (3)	1.71 (3)	2.6523 (19)	175 (2)

Symmetry code: (i) 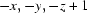.

**Fig. 1.**
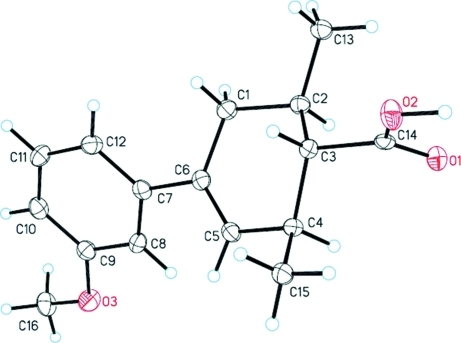
The molecular structure and atom numbering scheme.

**Fig. 2.**
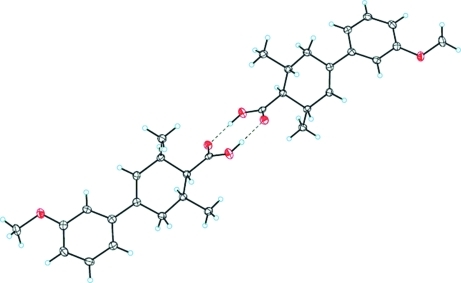
Hydrogen bonded dimer. Dashed lines represent hydrogen bonds. [Symmetry code: #1 -x,-y,-z+1.]

## supplementary crystallographic information

### Comment

The title carboxylic acid was prepared to study the interaction of the two enantiomers in the solid state. We have previously reported the structure of its precursor, which is achiral and forms hydrogen-bonded dimers (Xie *et al.*, 2007b). The chirality of the title compound is solely generated by the presence of the double bond in the cyclohexene ring (Xie *et al.*, 2004). The resultant racemate is made up of carboxylic acid RS dimers (Xie *et al.*, 2002, 2007a, 2008a,b). The structure and atom numbering are shown in Fig. 1, which illustrates the half-chair conformation of the cyclohexene ring. The torsion angles involving atoms C4, C5, C6, C1, and C2 are near 180°. The carboxyl group is almost perpendicular to the cyclohexene ring with an angle of 85.3° between the O1—C14—O2—C3 plane and the C1—C6 ring. The double bond between C5—C6 is not fully conjugated with the aromatic ring as shown by the C1—C6—C5 plane to benzene ring angle of 38.7°. Unlike other previously reported *para* substituted analogs and like other previously reported *meta* substituted analogs (Xie *et al.*, 2008b), the molecule also has a chiral axis due to the *meta* methoxy substituent on the aromatic ring.

Fig. 2 shows the hydrogen bonding scheme. Atom O2 acts as a donor in an intermolecular hydrogen bond to atom O1, producing an R22(8) ring (Bernstein *et al.*, 1995), thus creating a hydrogen- bonded dimer. There is no evidence to suggest that weak directional interactions interconnect the dimers. Hydrogen bond geometry is given in Table 1.

### Experimental

The title carboxylic acid was synthesized following the similar method reported by Xie *et al.*, 2002. Purified compound was recrystallized from hexane- dichloromethane as colorless plates (m.p. 415-417 K).

### Refinement

The data collection was carried out using synchrotron radiation (λ= 0.44280, diamond 111 monochromator, two mirrors to exclude higher harmonics) with a frame time of 2 second and a detector distance of 6.0 cm. A randomly oriented region of reciprocal space was surveyed to the extent of a hemisphere. Two major sections of frames were collected with 0.50° steps in φ and a detector position of -20° in 2θ. Data to a resolution of 0.84 Å were considered in the reduction. Final cell constants were calculated from the xyz centroids of 2804 strong reflections from the actual data collection after integration (SAINT, Bruker Analytical X-Ray Systems, Madison, WI, 2008). The intensity data were corrected for absorption (SADABS) (Blessing, 1995).

The space group Pbca was determined based on intensity statistics and systematic absences. The structure was solved using SIR-2004 (Burla *et al.*, 2005) and refined with SHELXL-97 (Sheldrick, 2008). A direct-methods solution was calculated, which provided most non-hydrogen atoms from the E-map. Full-matrix least squares / difference Fourier cycles were performed, which located the remaining non-hydrogen atoms. All non-hydrogen atoms were refined with anisotropic displacement parameters. The hydrogen atoms were placed in ideal positions and refined as riding atoms with relative isotropic displacement parameters with the exception of the hydroxyl hydrogen atom, which was refined for all parameters. The final full matrix least squares refinement converged to R1 = 0.0368 and wR2 = 0.1190 (F^2^, all data). The structure was found as proposed. The remaining electron density is minuscule and located on bonds.

### Crystal data

**Table:**

C_16_H_20_O_3_	*F*(000) = 1120
*M**_r_* = 260.32	*D*_x_ = 1.244 Mg m^−^^3^
Orthorhombic, *P**b**c**a*	Synchrotron radiation, λ = 0.44280 Å
Hall symbol: -P 2ac 2ab	Cell parameters from 2804 reflections
*a* = 11.032 (2) Å	θ = 2.3–15.3°
*b* = 7.8423 (17) Å	µ = 0.05 mm^−^^1^
*c* = 32.140 (8) Å	*T* = 100 K
*V* = 2780.7 (11) Å^3^	Plate, colorless
*Z* = 8	0.02 × 0.01 × 0.01 mm

### Data collection

**Table:**

Bruker Platform goniometer diffractometer	2173 independent reflections
Radiation source: synchrotron	1755 reflections with *I* > 2σ(*I*)
diamond 1 1 1	*R*_int_ = 0.050
Detector resolution: 83.33 pixels mm^-1^	θ_max_ = 15.3°, θ_min_ = 0.8°
ω and phi scans	*h* = −11→13
Absorption correction: multi-scan (*SADABS*; Bruker, 2007; Blessing, 1995)	*k* = −8→6
*T*_min_ = 0.999, *T*_max_ = 1.000	*l* = −38→26
8586 measured reflections	

### Refinement

**Table:**

Refinement on *F*^2^	Primary atom site location: structure-invariant direct methods
Least-squares matrix: full	Secondary atom site location: difference Fourier map
*R*[*F*^2^ > 2σ(*F*^2^)] = 0.037	Hydrogen site location: inferred from neighbouring sites
*wR*(*F*^2^) = 0.119	H atoms treated by a mixture of independent and constrained refinement
*S* = 1.11	*w* = 1/[σ^2^(*F*_o_^2^) + (0.0707*P*)^2^ + 0.0727*P*] where *P* = (*F*_o_^2^ + 2*F*_c_^2^)/3
2173 reflections	(Δ/σ)_max_ = 0.001
179 parameters	Δρ_max_ = 0.17 e Å^−^^3^
0 restraints	Δρ_min_ = −0.23 e Å^−^^3^

### Special details

**Table:**

Geometry. All esds (except the esd in the dihedral angle between two l.s. planes) are estimated using the full covariance matrix. The cell esds are taken into account individually in the estimation of esds in distances, angles and torsion angles; correlations between esds in cell parameters are only used when they are defined by crystal symmetry. An approximate (isotropic) treatment of cell esds is used for estimating esds involving l.s. planes.
Refinement. Refinement of *F*^2^ against ALL reflections. The weighted *R*-factor wR and goodness of fit *S* are based on *F*^2^, conventional *R*-factors *R* are based on *F*, with *F* set to zero for negative *F*^2^. The threshold expression of *F*^2^ > σ(*F*^2^) is used only for calculating *R*-factors(gt) etc. and is not relevant to the choice of reflections for refinement. *R*-factors based on *F*^2^ are statistically about twice as large as those based on *F*, and *R*- factors based on ALL data will be even larger.

### Fractional atomic coordinates and isotropic or equivalent isotropic displacement parameters (Å^2^)

**Table:**

	*x*	*y*	*z*	*U*_iso_*/*U*_eq_	
O1	−0.01213 (10)	0.06346 (16)	0.54893 (3)	0.0182 (3)	
O2	0.02685 (11)	0.22640 (18)	0.49329 (4)	0.0209 (3)	
H2O	0.018 (2)	0.122 (4)	0.4790 (8)	0.049 (7)*	
O3	0.19151 (12)	0.77788 (18)	0.76439 (3)	0.0250 (4)	
C1	−0.07178 (15)	0.5720 (2)	0.60649 (5)	0.0173 (4)	
H1A	−0.0730	0.6650	0.5857	0.021*	
H1B	−0.1403	0.5909	0.6258	0.021*	
C2	−0.09016 (14)	0.4018 (2)	0.58418 (5)	0.0152 (4)	
H2	−0.1031	0.3108	0.6055	0.018*	
C3	0.02669 (14)	0.3611 (2)	0.55976 (5)	0.0141 (4)	
H3	0.0446	0.4592	0.5409	0.017*	
C4	0.13603 (14)	0.3370 (2)	0.58944 (5)	0.0148 (4)	
H4	0.1288	0.2227	0.6030	0.018*	
C5	0.13641 (15)	0.4715 (2)	0.62290 (5)	0.0161 (4)	
H5	0.2066	0.4796	0.6399	0.019*	
C6	0.04532 (15)	0.5812 (2)	0.63059 (5)	0.0152 (4)	
C7	0.05757 (14)	0.7208 (2)	0.66193 (5)	0.0157 (4)	
C8	0.11780 (15)	0.6918 (2)	0.69955 (5)	0.0167 (4)	
H8	0.1500	0.5820	0.7054	0.020*	
C9	0.13098 (15)	0.8233 (2)	0.72858 (5)	0.0181 (4)	
C10	0.08441 (15)	0.9845 (2)	0.72076 (5)	0.0202 (4)	
H10	0.0930	1.0736	0.7406	0.024*	
C11	0.02467 (16)	1.0126 (3)	0.68315 (5)	0.0209 (4)	
H11	−0.0067	1.1228	0.6772	0.025*	
C12	0.01008 (15)	0.8828 (2)	0.65423 (5)	0.0189 (4)	
H12	−0.0324	0.9042	0.6291	0.023*	
C13	−0.20110 (15)	0.4090 (2)	0.55581 (5)	0.0194 (4)	
H13A	−0.2153	0.2962	0.5436	0.029*	
H13B	−0.1870	0.4920	0.5335	0.029*	
H13C	−0.2722	0.4433	0.5721	0.029*	
C14	0.01185 (14)	0.2031 (2)	0.53354 (5)	0.0137 (4)	
C15	0.25486 (15)	0.3418 (2)	0.56468 (5)	0.0183 (4)	
H15A	0.3235	0.3277	0.5837	0.027*	
H15B	0.2618	0.4515	0.5503	0.027*	
H15C	0.2552	0.2492	0.5442	0.027*	
C16	0.19283 (18)	0.8987 (3)	0.79772 (5)	0.0244 (5)	
H16A	0.2328	0.8483	0.8220	0.037*	
H16B	0.1094	0.9297	0.8050	0.037*	
H16C	0.2371	1.0010	0.7890	0.037*	

### Atomic displacement parameters (Å^2^)

**Table:**

	*U*^11^	*U*^22^	*U*^33^	*U*^12^	*U*^13^	*U*^23^
O1	0.0202 (7)	0.0155 (9)	0.0188 (7)	−0.0023 (5)	0.0004 (5)	−0.0007 (5)
O2	0.0300 (7)	0.0176 (10)	0.0153 (7)	−0.0046 (5)	0.0012 (5)	−0.0023 (5)
O3	0.0342 (8)	0.0229 (10)	0.0177 (7)	0.0040 (5)	−0.0082 (5)	−0.0049 (5)
C1	0.0154 (9)	0.0176 (13)	0.0190 (9)	0.0023 (7)	0.0011 (7)	−0.0024 (7)
C2	0.0133 (9)	0.0163 (12)	0.0161 (8)	0.0002 (6)	0.0005 (6)	0.0001 (6)
C3	0.0147 (9)	0.0130 (12)	0.0148 (8)	−0.0001 (6)	−0.0009 (6)	0.0004 (7)
C4	0.0126 (9)	0.0149 (12)	0.0169 (8)	0.0003 (6)	−0.0016 (6)	0.0007 (7)
C5	0.0159 (9)	0.0165 (12)	0.0159 (8)	−0.0020 (7)	−0.0018 (6)	−0.0007 (7)
C6	0.0162 (9)	0.0151 (12)	0.0142 (8)	−0.0016 (7)	0.0014 (7)	0.0018 (6)
C7	0.0119 (9)	0.0179 (13)	0.0171 (9)	−0.0020 (7)	0.0038 (6)	−0.0007 (7)
C8	0.0182 (9)	0.0138 (12)	0.0181 (9)	0.0009 (6)	0.0022 (6)	−0.0004 (6)
C9	0.0175 (9)	0.0198 (13)	0.0170 (8)	−0.0018 (7)	0.0005 (7)	0.0002 (7)
C10	0.0197 (10)	0.0204 (13)	0.0205 (9)	−0.0017 (7)	0.0028 (7)	−0.0063 (7)
C11	0.0228 (10)	0.0161 (13)	0.0239 (10)	0.0045 (7)	0.0012 (7)	−0.0020 (7)
C12	0.0172 (9)	0.0206 (14)	0.0189 (9)	0.0025 (7)	−0.0013 (7)	−0.0012 (7)
C13	0.0145 (9)	0.0199 (13)	0.0238 (9)	0.0016 (7)	−0.0021 (7)	−0.0031 (7)
C14	0.0074 (8)	0.0156 (13)	0.0181 (9)	0.0008 (6)	−0.0011 (6)	0.0011 (7)
C15	0.0151 (9)	0.0173 (12)	0.0225 (8)	0.0000 (7)	−0.0005 (7)	−0.0029 (7)
C16	0.0330 (11)	0.0235 (14)	0.0168 (9)	−0.0040 (8)	−0.0029 (8)	−0.0055 (7)

### Geometric parameters (Å, °)

**Table:**

O1—C14	1.230 (2)	C6—C7	1.493 (2)
O2—C14	1.317 (2)	C7—C12	1.396 (3)
O2—H2O	0.94 (3)	C7—C8	1.398 (2)
O3—C9	1.378 (2)	C8—C9	1.399 (2)
O3—C16	1.430 (2)	C8—H8	0.9500
C1—C6	1.508 (2)	C9—C10	1.387 (3)
C1—C2	1.528 (2)	C10—C11	1.394 (2)
C1—H1A	0.9900	C10—H10	0.9500
C1—H1B	0.9900	C11—C12	1.388 (3)
C2—C13	1.527 (2)	C11—H11	0.9500
C2—C3	1.543 (2)	C12—H12	0.9500
C2—H2	1.0000	C13—H13A	0.9800
C3—C14	1.508 (2)	C13—H13B	0.9800
C3—C4	1.549 (2)	C13—H13C	0.9800
C3—H3	1.0000	C15—H15A	0.9800
C4—C5	1.507 (2)	C15—H15B	0.9800
C4—C15	1.534 (2)	C15—H15C	0.9800
C4—H4	1.0000	C16—H16A	0.9800
C5—C6	1.346 (3)	C16—H16B	0.9800
C5—H5	0.9500	C16—H16C	0.9800
			
C14—O2—H2O	110.1 (16)	C7—C8—H8	119.8
C9—O3—C16	117.34 (15)	C9—C8—H8	119.8
C6—C1—C2	113.35 (14)	O3—C9—C10	124.50 (15)
C6—C1—H1A	108.9	O3—C9—C8	114.65 (16)
C2—C1—H1A	108.9	C10—C9—C8	120.85 (16)
C6—C1—H1B	108.9	C9—C10—C11	118.43 (16)
C2—C1—H1B	108.9	C9—C10—H10	120.8
H1A—C1—H1B	107.7	C11—C10—H10	120.8
C13—C2—C1	110.76 (14)	C12—C11—C10	121.27 (18)
C13—C2—C3	111.93 (13)	C12—C11—H11	119.4
C1—C2—C3	107.97 (13)	C10—C11—H11	119.4
C13—C2—H2	108.7	C11—C12—C7	120.36 (16)
C1—C2—H2	108.7	C11—C12—H12	119.8
C3—C2—H2	108.7	C7—C12—H12	119.8
C14—C3—C2	111.34 (13)	C2—C13—H13A	109.5
C14—C3—C4	109.16 (14)	C2—C13—H13B	109.5
C2—C3—C4	111.26 (13)	H13A—C13—H13B	109.5
C14—C3—H3	108.3	C2—C13—H13C	109.5
C2—C3—H3	108.3	H13A—C13—H13C	109.5
C4—C3—H3	108.3	H13B—C13—H13C	109.5
C5—C4—C15	110.53 (14)	O1—C14—O2	123.06 (15)
C5—C4—C3	110.86 (14)	O1—C14—C3	122.03 (14)
C15—C4—C3	110.06 (13)	O2—C14—C3	114.91 (15)
C5—C4—H4	108.4	C4—C15—H15A	109.5
C15—C4—H4	108.4	C4—C15—H15B	109.5
C3—C4—H4	108.4	H15A—C15—H15B	109.5
C6—C5—C4	125.23 (15)	C4—C15—H15C	109.5
C6—C5—H5	117.4	H15A—C15—H15C	109.5
C4—C5—H5	117.4	H15B—C15—H15C	109.5
C5—C6—C7	121.65 (15)	O3—C16—H16A	109.5
C5—C6—C1	120.98 (16)	O3—C16—H16B	109.5
C7—C6—C1	117.32 (14)	H16A—C16—H16B	109.5
C12—C7—C8	118.65 (15)	O3—C16—H16C	109.5
C12—C7—C6	120.89 (15)	H16A—C16—H16C	109.5
C8—C7—C6	120.46 (16)	H16B—C16—H16C	109.5
C7—C8—C9	120.42 (17)		
			
C6—C1—C2—C13	−172.40 (14)	C5—C6—C7—C8	−39.5 (2)
C6—C1—C2—C3	−49.53 (18)	C1—C6—C7—C8	142.90 (16)
C13—C2—C3—C14	−52.46 (19)	C12—C7—C8—C9	−0.6 (2)
C1—C2—C3—C14	−174.62 (13)	C6—C7—C8—C9	178.94 (15)
C13—C2—C3—C4	−174.47 (15)	C16—O3—C9—C10	8.2 (2)
C1—C2—C3—C4	63.37 (17)	C16—O3—C9—C8	−171.22 (15)
C14—C3—C4—C5	−166.45 (14)	C7—C8—C9—O3	179.71 (14)
C2—C3—C4—C5	−43.19 (19)	C7—C8—C9—C10	0.2 (2)
C14—C3—C4—C15	70.95 (17)	O3—C9—C10—C11	−179.78 (16)
C2—C3—C4—C15	−165.78 (14)	C8—C9—C10—C11	−0.4 (2)
C15—C4—C5—C6	132.37 (18)	C9—C10—C11—C12	0.9 (3)
C3—C4—C5—C6	10.1 (2)	C10—C11—C12—C7	−1.3 (3)
C4—C5—C6—C7	−174.49 (16)	C8—C7—C12—C11	1.2 (2)
C4—C5—C6—C1	3.0 (3)	C6—C7—C12—C11	−178.39 (15)
C2—C1—C6—C5	17.8 (2)	C2—C3—C14—O1	−60.2 (2)
C2—C1—C6—C7	−164.57 (14)	C4—C3—C14—O1	63.06 (19)
C5—C6—C7—C12	140.02 (18)	C2—C3—C14—O2	120.03 (15)
C1—C6—C7—C12	−37.5 (2)	C4—C3—C14—O2	−116.76 (15)

### Hydrogen-bond geometry (Å, °)

**Table:**

*D*—H···*A*	*D*—H	H···*A*	*D*···*A*	*D*—H···*A*
O2—H2O···O1^i^	0.94 (3)	1.71 (3)	2.6523 (19)	175 (2)

Symmetry codes: (i) −*x*, −*y*, −*z*+1.
